# Cross-cultural adaptation of the Arabic Positive and Negative Syndrome Scale in schizophrenia: Qualitative analysis of a focus group

**DOI:** 10.1177/1363461519850345

**Published:** 2019-05-27

**Authors:** Iman Amro, Suhaila Ghuloum, Ziyad Mahfoud, Mark Opler, Anzalee Khan, Samer Hammoudeh, Yahya Hani, Arij Yehya, Hassen Al-Amin

**Affiliations:** Weill Cornell Medicine – Qatar; Rumailah Hospital, Qatar; Weill Cornell Medicine – Qatar; CSO-Prophase LLC, USA; NeuroCog Trials, USA; Nathan S. Kline Institute for Psychiatric Research, USA; Weill Cornell Medicine – Qatar; Rumailah Hospital, Qatar; Weill Cornell Medicine – Qatar; Weill Cornell Medicine – Qatar

**Keywords:** Arabic translation, cultural validity, focus group, PANSS, psychiatric assessment, psychosis

## Abstract

As part of a project to translate and validate scales used in the diagnosis and treatment of Arab patients with schizophrenia, this study aimed to explore the experience of clinical research coordinators (CRCs) while administering the Arabic version of the Positive and Negative Syndrome Scale (PANSS) on Arab schizophrenia patients. We previously reported that the Arabic version of PANSS is a valid and reliable tool to assess Arab patients with schizophrenia. Five CRCs and the principal investigator attended focus group discussions on cultural issues in administering the PANSS. A thematic analysis approach was utilized for data coding and analysis. The results identified issues related to the translation of the instrument, the structure of the interview, the cultural sensitivity of some questions, and the procedures for rating items of the PANSS. Qualitative analysis also identified four main themes relevant to clinical assessment of patients from Middle Eastern cultures: religion, beliefs and values, gender, and semantic expressions. In conclusion, researchers or clinicians administering the PANSS scale interview in Arabic should be trained to consider the roles of local dialects, familiarity with abstract thinking, religion, and social constructs when assessing psychosis.

## Introduction

**S**chizophrenia is a chronic mental illness characterized by positive symptoms (such as hallucinations, delusions, and disorganized thinking), negative symptoms (like avolition, alogia, affect flattening, and lack of motivation), and cognitive deficits in working memory, verbal skills, and attention. The lifetime prevalence of schizophrenia is 0.4–1% of the populations worldwide, but there are regional, cultural, and racial variations (Jablensky et al., 1992; Kirkbride et al., 2006, 2007; Picchioni & Murray 2007). The few studies that have assessed the prevalence of schizophrenia in the Arab countries have found that the rates are similar to those reported in other populations around the world (Abou-Saleh, Ghubash, & Daradkeh, 2001; Ghuloum, Bener, & Abou-Saleh, 2011; Karam et al., 2006). Schizophrenia is usually diagnosed after a clinical interview and based on the International Statistical Classification of Disease and related health problems (ICD-10; WHO, 2010) or the Diagnostic and Statistical Manual (DSM) of mental disorders (American Psychiatric Association, 2000), which are based on field studies and expert opinions. These studies were conducted mainly in Western countries like the USA and Europe. Such diagnostic interviews like the Structured Clinical Interview for DSM-IV-SCID (First, Spitzer, Gibbon, & Williams, 2002) and the Mini International Neuropsychiatric Interview for Schizophrenia and other Psychotic Disorders-MINI (MINI-6; Amorim, Lecrubier, Weiller, Hergueta, & Sheehan, 1998) are mostly used in research to minimize clinician bias. The MINI has been translated and tested within the Arabic context (Ghanem, Gadallah, Meky, Mourad, & El Kholy, 2009; Kadri et al., 2005) and it follows the DSM, fourth edition, text revision (DSM-IV-TR) criteria (American Psychiatric Association, 2000) to confirm the diagnosis through a semi-structured interview.

Other psychiatric scales assess the severity of psychosis and can be used to monitor the effectiveness of the treatment. Examples of these tools include the Brief Psychiatric Rating Scale (BPRS) (Overall & Gorham, 1962) and the Positive and Negative Syndrome Scale (PANSS) (Kay, Fiszbein, & Opler, 1987). PANSS is a standardized instrument to assess symptom severity in schizophrenia and is widely used in clinical research across various regions and cultures. It includes 30 items that generate a total severity score and three subscales of mutually exclusive items measuring positive symptoms, negative symptoms, and general psychopathology, including anxiety, depression, insight, etc. The PANSS ratings are based on the Structured Clinical Interview for the PANSS (SCI-PANSS). Since its publication in 1987, PANSS has been translated into more than 30 official languages (Opler & Ramirez, 1998). Recently, we quantitatively validated the Arabic version of PANSS in Arabs with schizophrenia (Yehya et al., 2016, 2017).

Earlier studies have suggested that schizophrenia may be viewed in very similar ways in many cultures and that the degree of the similarity in symptoms and recognition of illness across cultures increases with disease severity (White, 1982). On the other hand, the measurement of symptoms associated with schizophrenia has been criticized for the lack of attention to cultural variation (Bhui et al., 2003). For example, studies comparing the manifestations of schizophrenia in different cultures (Jablensky et al., 1992; Sartorius et al., 1986) have reported that in Western countries, schizophrenia patients showed higher frequency of depressive symptoms, primary delusions, and thought insertion and broadcasting, while in non-Western countries, they showed more visual and directed auditory hallucinations. There is also evidence that inadequate translation and adaptation of an instrument can affect scale reliability (Berkanovic, 1980; Zandi, Havenaar, Laan, Kahn, & Brink, 2016). There are well-established protocols for the translation and cross-cultural adaptation of mental health instruments to ensure semantic, conceptual, and technical equivalence between the original and adapted versions (Simonsen & Mortensen, 1990). Qualitative research methods can be used to explore the self-perception of symptoms and social phenomena to establish cross-cultural equivalence (Malterud, 2001).

This study explored the experiences of clinical research coordinators (CRCs) involved in administering the PANSS using qualitative thematic analysis after focus group discussions. The main themes and challenges faced by the CRCs during the validation project were categorized and analyzed, together with the approaches utilized to address them.

## Methods

### Study design

This is a qualitative research study that was part of the project to translate, culturally adapt, and validate the Arabic version of PANSS in patients with schizophrenia in Qatar. This study adopted the qualitative approach using focus group discussions. This method is well known for the flexibility it provides in exploring the participants’ experience (Miles & Huberman, 1994). Furthermore, focus group discussions have proved to be useful in mental health research, by providing qualitative information for both qualitative and quantitative research designs (Room, Janca, Bennett, Schmidt, & Sartorius, 1996).

### Study setting

Qatar is one of the six countries that belong to the Gulf Cooperation Council (GCC) in the Arabian Peninsula. It is a rapidly growing state that includes immigrants from different nationalities and cultures. The total population in Qatar is estimated to be about 2.5 million, of which over 2 million are expatriates. The Arabs, including Qataris, who are the most settled group in Qatar, represent about 27% of the total population: 12.3% are Qataris, and 14.7% are Arabs from different nationalities (Qatar Statistics Authority, 2015). This project was conducted between February 2013 and November 2014, and was a collaborative effort between the Department of Psychiatry at Rumailah Hospital, Hamad Medical Corporation (HMC) in Qatar and Weill Cornell Medicine – Qatar. The study protocol was approved by the IRB committees of both institutions (Protocol number: 11129/11; Research number: NPRP 4-268-3-086) and was funded by the Qatar National Research Fund (QNRF). The target population for the validation project was Arab schizophrenia patients attending the Psychiatry Department. The latter is the only psychiatric facility in Qatar. It has 10 outpatient clinics with about 120 visits per weekday, and four inpatient wards (70 beds) with 90–95% occupancy. The majority of the inpatients are admitted with acute psychotic symptoms, and about 25% of the outpatients have the diagnosis of schizophrenia.

A previously published paper provides details on the quantitative study for which the PANSS was translated (Yehya et al., 2016). Briefly, the original PANSS was translated to Arabic using the back-translation method for cross-cultural adaptation of psychiatric scales (Prince, 2008). Three bilingual psychiatrists and two professional translators were involved in this translation procedure. During this adaptation process, the feedback from a pilot sample (10 schizophrenia subjects and 10 normal controls) was also incorporated to prepare the final Arabic version. The feedback of the CRCs (described below) was also integrated into the translation and training for the administration of PANSS to Arab patients. The copyright holder (Multi Health Systems Inc., Ontario, Canada) of PANSS approved the final version of the English back-translation of PANSS. The Arabic version of the MINI-6 for schizophrenia module K (Amorim et al., 1998) was used to confirm the diagnosis of schizophrenia in the eligible subjects involved in the study, which included 101 Arab patients diagnosed with schizophrenia and 98 Arabs with no mental disorders.

### Focus group participants

Five clinical research coordinators (CRCs) who were involved in the recruitment process, and the study moderator who is the principal investigator for the validation project, met to explore their experiences in a focus group. This meeting was semi-structured and chaired by the moderator. All participants were bilingual English and Arabic speakers of different Arabic dialects. They had diverse health/medical backgrounds (medical doctors, psychologist, nursing, and other health professionals), with varied experience in mental health research.

The participants were all trained in the administration of PANSS and attended an intensive training course held by the PANSS Institute. The participants were also trained to use the MINI-6 by Dr. Sheehan and other psychiatrists on the research team. All the CRCs were supervised on at least five cases before they started assessing subjects on their own. These focus group discussions were held after each of the CRCs had conducted at least 20 assessments with the Arabic PANSS. The group participants knew each other well and had worked together on this project for several months before these focus group discussions. The focus groups were part of the translation and training procedures for collecting the data for the quantitative analysis and they were approved as part of the overall research proposal and did not require a separate consent process.

### Focus group discussions

The CRCs were invited to take part in two focus group discussions; each session lasted approximately 90 minutes. Both languages (Arabic and English) were used during the discussions, which were audio-recorded and transcribed by the first author. Semi-structured discussions using prompts were employed to elicit the insight and experiences of the CRCs about the PANSS scale administration and the challenges they faced. Examples of the questions utilized by the moderator are listed in Appendix I. These questions and others were generated by the moderator and the other bilingual practicing psychiatrists who were involved in the translation and validation of the Arabic version of PANSS. Particular attention was paid to determine whether there were domains that should be added to, removed from, or modified in the Arabic version of the scale. The moderator encouraged the CRCs to elaborate on issues that had an impact on the process of scale administration and ratings. They were also invited to describe scenarios from the clinical practice where translation or cultural issues were raised when administering the PANSS.

### Data analysis

A thematic analysis approach was utilized to categorize the emerging themes, supported by quotes taken from the focus group discussions. The following six steps were followed for this thematic analysis: 1) all transcribed materials and recordings were revised and revisited many times to understand the data and note the preliminary ideas; 2) the transcripts were read through to get a general flow of the material as a whole, and then initial observations from the dataset were coded; 3) the initial comments and codes were analyzed to identify the possible organizations of the themes and subthemes; 4) whenever ideas shared the same concept, they were allocated to the same theme and potential subthemes. At this stage, we were able to refine the analysis and organize the thematic chart, which led to sub-themes being identified and fed back into the main themes; 5) the organized themes and subthemes were then defined according to the overall understanding of their cultural significance; 6) in the last phase, the emerging themes and subthemes were supported by quotes from the participants and divided according to their relevance to the goals of this research study. The thematic analysis approach adopted here allowed us to identify items that were not well understood or were misinterpreted from the scale’s original intent. The first author carried out the analyses under the supervision of the principal investigator and the biostatistician in the team, and all data were anonymous and treated confidentially. In order to support confirmability and reduce bias, the data analysis involved research members who did not participate in the translation or in the focus group discussions. The credibility of this analysis was enhanced by including feedback from the pilot study on 10 patients and 10 healthy controls from the staff at the department.

## Results

The results section is divided into two parts: 1) general observations and findings of the SCI-PANSS and the rating criteria; and 2) cultural themes that emerged from the focus group discussions on the assessment processes.

### PANSS interview and rating

The CRCs elaborated extensively on the details related to the SCI interview and the PANSS criteria for rating. Table 1 presents the main relevant areas of discussion. All CRCs agreed that although training and practice helped them to process and deal with these issues, many of these matters still had to be approached individually throughout the study. The CRCs agreed that the formal Arabic translation of PANSS is not always commensurate with the different Arabic dialects. For example, the official Arabic word for “delusion” in not commonly used in daily language. Frequently, they adjusted the dialogue during the interview, taking into consideration the patients’ knowledge and understanding, the local culture, education level, mental illness perception, and Arabic dialect used. Adjusting and replacing certain terms is something that the CRCs used to do instantly while interviewing the subjects. This issue was difficult to manage during the proverbs section of the PANSS where most of the proverbs (when translated exactly) were not very well known to patients. Thus, to fit with the subjects’ culture many of the sayings listed in PANSS were changed in the Arabic translations and interview, with equivalent local phrases corresponding to the four levels of difficulty as per Appendix B of the original SCI-PANSS. For example, the proverb “Carrying a chip on your shoulder” was replaced with “Coming with trouble on his face,” which in local Arabic dialect reflects the same meaning. During the early stages of the study, the CRCs questioned the importance of the instructions and introductory paragraph of the SCI-PANSS, wondering if this is essential for the rating of psychosis. They also discussed the concept of structured vs. semi-structured technique when interviewing the subjects and whether they should be very strict when administering the questions of the SCI-PANSS. They also agreed that there was overlap or repetition in some of the questions, such as the example presented in Table 1, since both questions ask the same thing. However, one CRC indicated that “Question 14 comes under the delusions section while 126 comes under grandiosity section …” which might indicate different processes of thinking.

**Table 1.** Issues related to the PANSS interview and ratings.

Issues	Illustrative quotes from focus group participants
Language and translation issues	“Lots of sub-cultural terms are presented in the proverbs section … sometimes we don’t know whether the difficulty in understanding is because of the abstract way of thinking or the patient can’t understand what the proverb means … as most of them are difficult …” Another CRC suggested that “… sometimes I ask the patient to give us one proverb from their culture that he knows and explain it to us …” which is often difficult for patients to do.
Introductory/instruction part	“… Usually, I skip this part of the introduction and the instruction as I have started talking to the patient earlier when introducing the study, and we’ve already built the rapport …”
Structured vs. semi- structured interviews	“… I’ve noticed something about the SCI … within each group of questions there is a clear flow … but within each section there is not … you feel each question presents a different idea … for instance, you ask about the date to check the orientation, and after that you ask something different …”
Overlapping and repetitive questions	All CRCs agreed that there is some overlap in many of the questions, and it is not very clear when administering the scale. For instance: “… What is the difference between Question 14 and Question 126 in the SCI-PANSS …?” Question 14: “Can you read other people’s minds?” Question 126: “Do you have extrasensory perceptions (ESP), or can you read other people’s minds?”
Rating criteria	CRCs agreed “… reading all the potential answers made it easier to rate … sometimes I skip this step, as I became more familiar with the scale and the rating criteria …”
Sensitive questions	The group highlighted that all questions from 136 to 142 in the SCI-PANSS “are considered culturally not appropriate” as these questions pertain to religion. One CRC said that “… All these are related to religious beliefs, especially the one asking … perhaps you consider yourself God … this is considered a taboo in the Arab culture and Muslim community …”

Several prompts about the rating criteria were presented during the discussion. For example, “Do the evaluation criteria and the scores of 1 to 7 make sense to you? Do you always need to read all the options before you rate or is it easier to focus on two ratings each time?” The CRCs agreed that, at the beginning, reading all the potential answers made it easier to rate. However, with time, they were able to skip this step, as they became more familiar with the scale and the rating criteria. The number of items to read before finalizing the score was always dependent on each case and the complexities of the history and psychiatric manifestations. Another issue related to the interview is how to present the questions on culturally sensitive issues like religion, sex, etc. All CRCs agreed that these always needed to be introduced properly during the interview, otherwise “subjects might feel offended.” One CRC indicated that some of the subjects answered the culturally sensitive questions smoothly without any problems, especially the one about being a prophet because the patient was grandiose.

### Specific cultural themes

The four main cultural themes extracted from the analysis of the focus group discussions were religion, local faith and values, gender, and semantic constructs. Table 2 presents the topics with the corresponding illustrations. These themes and subthemes were supported by the discussions of the focus group, but only a sample of the main representative quotes is listed in the table.

**Table 2.** Cultural themes and illustrative quotes from focus group participants.

Themes	Sub-themes	Quotes
Religion	Talking about God Asking about suicide Having special powers	“In Arab culture I find talking about being God means that you are talking religious taboos …” “Some patients will give you that shocked face … Like how do you even ask me this kind of question, and they say it in different ways, verbally or emotionally …”
Beliefs	Delusions or possessions Strange or unusual experiences	“In the Arab community, they are still convinced that psychiatric illness [madness] is still somehow related to Jinn and most of the patients went to see the Motawwa [healer], so the latter can deal with the Jinn or magic before they come to the hospital …”
Gender	Male-dominant culture Segregation between males and females The unfortunate female being mentally ill	“I came across more than one female patient who refused to be recruited by a male research coordinator …” “Some of the female patients don’t follow up with their appointments, and they are struggling somehow because of the social stigma …”
Semantics	Words and proverbs that are not very familiar in Arabic dialects Concepts that have no synonyms in the Arabic language and local culture Rating criteria and the intended meaning of certain terms	“The words mayor and governor are not used in the Arabic language and are culturally not applicable …” “The word hallucination is used and understandable in Arabic but not delusions, which makes it very difficult for us as clinical researchers to explain it and make the effort to use familiar words that are easy and used within the patient’s dialect and accent …” “As we all come from different backgrounds, speaking different Arabic dialects, standardizing the way people talk will help; I mean to standardize in our head the way that we have to explain the question and proceed with the patients’ assessment …” “We don't use the Arabic word to reflect depression; instead, we always describe the state of depression by using somatic terms to explain the state or express what we feel …”

#### Religion

Qatar is an Arabic country where Islamic religion is the central pillar of cultural, social, and even governmental principles. The CRCs agreed that some of the questions in the PANSS might be “culturally sensitive” and need to be adapted to the local culture, particularly those related to religion. They also discussed the effect of the religious beliefs on the subjects’ views of the illness, its course, and outcome. For instance, when asking the subjects whether they believed in God or consider themselves prophets, both subjects and CRCs felt uncomfortable; and such questions were met with resistance as if they were unacceptable. The CRCs noted that most patients reacted negatively and at times even refused any question that might increase their “bad deeds,” just because it’s “haram” (religiously forbidden) according to the faith of most Muslims. As discussed above, the CRCs agreed that tactfully introducing the questions related to religion as routine questions that are asked to all patients generally helped in easing the apprehension of the subjects interviewed and facilitated getting adequate responses from patients.

#### Faiths and values

It is well documented that Arabs firmly believe in the existence of supernatural forces such as “Jinn,” magic, and the evil eye. The CRCs elaborated on certain values amongst the participants that can have a significant impact on the delivery of PANSS. For instance, it is evident that most of the Arab communities are convinced that:

… Psychiatric illness is still somehow related to Jinn, and most of the patients would go to see the “Motawwa” [local healer] or other traditional healers to perform certain rituals and exorcise Jinn … if this repeatedly fails then families of patients would seek medical or psychiatric help …

Another example: “most of our patients have used the cultural rituals at some point, including the amulet writers who produce amulets to avoid the evil eye, before they seek professional help.” All group members noted that patients always attribute all their symptoms to spiritual values and that the evil eye has an effect on the way they think and behave in their normal daily lives. These shared values made it difficult to decide when such thoughts and behaviors are part of their cultural norms or are related to their psychotic features. The CRCs added that the training and initial supervision helped them in deciding the proper rating of the PANSS items where the scores have a wide range from 1 (absent) to 7 (extreme) while taking the cultural context into consideration.

#### Gender

Gender and social differences in how the patients perceive the cause of mental illness, as well as the way they deal, cope with, and treat it, have been observed by the CRCs. For example, compared to men, it is more common among female patients to cite the possession of “Jinn” and the evil eye as the cause of the illness, and generally they are always the first to seek help from traditional healers. The CRCs also observed that most female patients refused to be recruited by a male CRC, and, to a lesser extent, male patients also refused to be enrolled by a female CRC. The CRCs also noted that families tend to shield women more and to not bring them for treatment or proper assessment because, in Arabic culture, women are “the less fortunate,” and for them to be diagnosed with psychiatric illness means they will suffer more from social stigma for the rest of their lives. The CRCs were concerned that the aforementioned issues might affect the recruitment of females, the evaluations of the caregivers, and the reports of patients themselves, which ultimately might affect the ratings of the PANSS items. In fact, we had more males with schizophrenia in the quantitative study of PANSS (Yehya et al., 2016).

#### Semantic construct

One of the prominent themes that emerged in this study was the formal translation of the scale and the different Arabic dialects used by the CRCs and patients. Although the formal Arabic language is the official language in all Arab countries, we know that each country has its subculture and its own daily spoken dialect of Arabic. The formal language is commonly used in official settings. The differences in spoken dialect, communication methods, and presentation of psychiatric symptoms might also impact the administration and the ratings of this scale. Similarly, it was debated amongst the group whether Arabic language translation has captured some of the English concepts in the scale or not. For instance, when translating delusion and depression into the Arabic language, CRCs tend to use somatic terms to explain the state of it. Furthermore, the word “madness” is used to reflect that one patient has a psychiatric illness, without specifying any medical terminology to indicate the type of mental illness: “We do explain the state of mania without saying a term to reflect mania in Arabic …” Also, there are Arabic words that have different meanings based on the dialect spoken in each country. For example, the Egyptian dialect is entirely distinct from the one spoken in the GCC countries: “One day I observed one of my colleagues, there was a clear miscommunication, caused by the different dialects between her and the patient …”

All the CRCs were trained on using the English version of PANSS as well as the Arabic one. It was obvious that they preferred to use the English language when rating the PANSS. Consequently, this affected the scale administration, especially with respect to how to capture the intended meanings of certain terms and whether they are available in the Arabic language or not. As one CRC put it, “I think in English, ask in Arabic, then I will translate what the patient said, and then I rate …”

## Discussion

The aim of this qualitative study was to explore and identify the main socio-cultural themes encountered during the administration and rating of the Arabic PANSS. The first set of issues is related to the SCI-PANSS interview: formal Arabic vs. local dialects, developing a rapport with psychotic patients, the structure of the interview, understanding what appear to be repetitive inquiries, introducing sensitive questions, and the rating process (Table 1). The second set of themes is mostly inherent in the Arabic culture of the Middle East and North Africa (MENA): general religious/cultural beliefs, gender issues, and the variations in the Arabic constructs of the psychopathology in schizophrenia (Table 2). Most of the matters in the first set are usually related to the experience of CRCs and can be addressed adequately with proper training and supervision. These matters are essential during the processes of cultural adaptation, as they might affect the ratings and thus compromise the reliability and validity of PANSS across cultures. These measures were very comparable between the Arabic PANSS and the other versions of PANSS (Hallit, Obeid, Haddad, Kazour, & Kazour, 2017; Yehya et al., 2016, 2017). The following sections will focus mainly on the cultural themes.

Previous studies that explored the cultural issues in mental health in the Arab region reported that it is common for people to believe in supernatural possession—Jinn, the evil eye, and sorcery (Al-Krenawi & Graham, 1997; Al-Krenawi, Graham, & Kandah, 2000)—and many believe that these are the causes behind mental illness. The existence of supernatural forces has been mentioned in the Quran (the Holy book of Islam), and some Arabs firmly believe that these forces can be the cause of mental illnesses, and that these beliefs about the supernatural cause of mental illness are not merely due to cultural influences (Ally & Laher, 2008). However, a comparative study of patients in Pakistan and Saudi Arabia suggested that ethnic and cultural factors are reflected in the schizophrenia symptoms, even if both populations adhere to the same religion (Ahmed & Naeem, 1984). Participants in our study shared similar views, as they tried to focus on the root of the problem as defined by the Arab culture (as opposed to Islam). It is well documented that such beliefs leave the patients suffering for a long time before they reach the point of seeking the proper psychiatric treatment (El-Islam, 2008). Others argue that Islam as a religion has a positive impact on Muslim patients; for example, Islam gives Muslims a unique set of behaviors, ethics, and social values, which help them to develop adaptive strategies and strong family ties to deal with different life situations (Sabry & Vohra, 2013). It is worth adding that families are the ones usually seeking help from the religious figures or traditional healers. In communities such as Qatar and Kuwait, it has been demonstrated that families show great interest in caring for, and being part of decisions on behalf of, their sick ones (Harding & Curran, 1978). Other cultures have also demonstrated the relevance of family ties on the assessment and treatment of patients with schizophrenia (Lee, Yamada, Kim, & Dinh, 2015; Villares, Redko, & Mari, 2017). Thus, it is important for the teams using these scales in various cultures to decide during the training of the raters what is considered a normal versus pathological variant according to the local cultural norms. This is very relevant to PANSS, where rating on each item ranges from 1–2 as normal with graded intensity, up to 7 as extremely abnormal. In addition, several items in the PANSS mandate the input of the primary caregivers (family and staff) and the teams should be trained on how to address the impact of the cultural context on the ratings of these items.

Regarding the gender theme, Arabic societies are generally male-dominant. They have been described as “conservative,” and communication between the genders is not as open as in Western societies (Hawamdeh & Raigangar, 2014). Furthermore, studies have shown that there are gender differences in symptomatology among mentally ill patients (Versola-Russo, 2006). Social differences and gender issues are also commonly described in the literature on Arab culture (Al-Krenawi & Graham, 1999). A study by El-Islam and Abu-Dagga (1990) showed that education level had a significant influence on how men and women perceive the cause, treatment course, and outcome of their illnesses. Seeman, Seeman, and Sayles (1985) stated that gender differences might be attributed to the increased levels of stress due to the high expectations from males vs. the more social protective factors in females. In addition, some practices are part of local traditions that are also reinforced by the health care providers, rather than by religious taboos. For example, women usually ask for a female health care provider, and they don’t easily share personal issues. They tend to avoid eye contact and speak in a low tone with male physicians. On the other hand, and depending on their culture and religion, the clinicians themselves might reciprocate the women’s behaviors or perceive them as unwilling to engage in the interview. It is also a common practice in the GCC to match the local Arab women with female health care providers, although this is not mandated by the Islamic religion. Thus, these gender differences, together with their impact on recruitment and rating of PANSS items, should be taken into consideration during the research plans and training of raters. Such initial focus group discussions with caregivers, patients, families, and raters can improve the reliability and validity of the PANSS ratings.

Local studies have emphasized that mental health measurements and interventions need to be culturally adapted to the Middle Eastern context (Al-Krenawi et al., 2000). In this respect, we can add that a unique aspect characterizes the Arabic language, where the formal written version differs substantially from the spoken vernacular. There are also major and substantial differences that exist within the different Arabic regions and subcultures, for example between Bedouin and sedentary speech, the countryside and major cities, religious groups, men and women, as well as young and old (Bassiouney, 2009). Arabic speakers are often able to modulate the way they speak based on the circumstances, which can clearly impact the way patients express their clinical symptoms. For example, Nydell (1987) pointed out that Arabs use many repetitions of phrases and themes, along with exaggerated reports and descriptions, to stress the importance of what is being said. It was noted that Arabic patients who lack the knowledge of the Arabic medical jargon could change the way they express their emotions and psychological states. Thus, in Arab communities, the communication style and level of knowledge of patients should be taken into account when assessing how they express their symptoms and feelings. The impact of such themes on the assessment and diagnosis of patients has also been described in other countries. For example, in African Americans, evidence showed that a lack of cultural understanding, as well as racial differences in the presentation of psychiatric symptoms, was associated with an over-diagnosis of schizophrenia patients (Barnes, 2004). Other studies have demonstrated the benefits of culturally adapted assessments (Zandi et al., 2016) and interventions in patients with psychosis, especially on the ratings of symptom severity (Degnan et al., 2017; Raffard et al., 2015). Another important observation by the CRCs is the lack of clear semantic terms for the psychiatric symptoms among patients, e.g., words like depression, mania, delusion, etc. (Table 2). Instead, patients tend to use somatic terms and expressions and to seek medical treatments without insight into their possible relationship to their psychiatric disorder. This was also supported by the factor structure analysis of the Arabic version of PANSS, which showed very similar components to other culturally validated PANSS, except for the item on somatic concerns, which was factored as a separate component and was not associated with the other components of psychotic or depressive symptoms (Yehya et al., 2017).

There are some limitations that might affect the outcomes and should be controlled for in future studies. In the quantitative studies (Yehya et al., 2016), we elaborated on some of these limitations, e.g., sample size, gender differences, using formal Arabic translations, and the impact of level of education of participants. Other limitations that are more relevant to this qualitative study include the fact that the majority of the Arabs are Qataris in this sample, and that as such, the various Arabic sub-cultures and dialects are not represented well enough to generalize the results to all Arabs. Larger studies using local dialects in specific Arabic countries with homogeneous local CRCs might further our understanding of the impact of Arabic sub-cultures on PANSS content and ratings. Another limitation is the lack of qualitative thematic analysis from interviews with the patients themselves and their families, which is important to better understand the main socio-cultural themes related to the assessment of patients with schizophrenia. Larger initial piloting of patients and caregivers can provide feedback for refining the translations and for training raters to administer the PANSS.

## Conclusion

The results of this qualitative analysis of the issues encountered when administering the Arabic version of PANSS to Arab patients with schizophrenia highlighted several matters related to the conduct of the interview itself, the semantic translations, the clinical relevance of some of the items, and the process of rating the 30 items in PANSS. In addition, the analysis confirmed several themes that every research team needs to address adequately in the Arabic population, including the religious convictions that might overlap with some psychotic features, the gender differences in expressing the various symptoms, and how the communication styles can affect the assessment of psychopathology in Arab patients with schizophrenia.

## Appendix I

Questions used by the facilitator of the focus group discussions:

1. What does the group think about the Arabic translation per se and did the patients comment on specific translational issues?
2. How did you handle the different Arabic sub-cultures when administering the Arabic SCI-PANSS?
3. What questions needed further elaborations or explanations during the interview?
4. What were the advantages and challenges encountered during the interview using Arabic SCI-PANSS?
5. What themes or topics were difficult to engage patients with or to assess their responses?
6. When evaluating the patients, what cultural matters did you encounter and how did you handle them? What were the reactions of the patients to these matters?
7. When using the item descriptions in the PANSS, what challenges did you face?
8. When rating the different items in PANSS, did you have to read all the descriptions of each rating after each assessment?
9. How did you handle the items that require the input of the primary care givers? Did you rely on the family members or the staff involved in patients’ care?
10. Did the training prepare you to handle the challenges you encountered during the assessment and rating procedures? Any suggestions for further training?
